# The Constituent Year Effect: Relative Age Disparities in Australian Masters Track and Field Athletic Participation

**DOI:** 10.3390/sports6040167

**Published:** 2018-12-08

**Authors:** Nikola Medic, Jasmine Lares, Bradley W. Young

**Affiliations:** 1 Centre for Exercise and Sports Science Research, Edith Cowan University, Joondalup 6027, Australia; jlares@our.ecu.edu.au; 2 Faculty of Health Sciences, University of Ottawa, Ottawa K1N 6N5, Canada; byoung@uottawa.ca

**Keywords:** masters athletes, sport motivation, sport participation, aging, relative age effect

## Abstract

The constituent year effect, a source of relative age disparities, in masters sport has been demonstrated mainly amongst North American samples. Thus, the purpose of this study was to examine whether a participation-related constituent year effect exists among athletes (*n* = 6492) competing in Australian Masters Athletics competitions between 2000 and 2014. The results indicated that a participation-related constituent year effect was observed as the likelihood of participating was significantly higher for masters athletes in their first and second constituent year of any five-year age category (*p* < 0.0001) and was lower when they were in the fourth or fifth constituent year. The results also indicated this effect is influenced by gender and age. Specifically, the effect was significant for both male (*p* < 0.0001) and female (*p* < 0.001) masters athletes; as well during the third, sixth, seventh, and eighth + decades of life (all *ps* < 0.001). These data demonstrate that despite masters sport being an avenue for promotion of participation and overall health, there is potential for improving how competitive organizational strategies are implemented given the recurring intermittent patterns of participation associated with five-year age brackets which are likely to compromise benefits.

## 1. Introduction

Considerable evidence exists to support the link between regular physical activity, including sport and health promotion in terms of physiological [1] and psychological [2] benefits. Nevertheless, participation rates in physical activities, such as sport remain generally poor, and this trend is particularly evident in older adult cohorts where decreasing levels of physical activity participation are typically noted compared to younger age cohorts [3]. Contrary to this trend, however, is the growing recognition and appeal of participation in Masters-level sport which indicates an increasing number of National- and Internationally-based competitions for masters athletes since the inception of these events in the 1970s [4,5]. Masters athletes are individuals who are typically over 35 years old and who have either maintained or at some later time in their life resumed their sport participation [6]. The status of a masters athlete is determined by each sport governing body and is based on the age at which peak performance (i.e., the open world record) occurs in a respective sport [7,8]. For instance, the Masters designation begins at 25 years of age for swimming, at 35 years of age for track and field, and at 50 years of age for golf.

In masters sports, a common motivational approach to organizing competitive events is to distribute individuals within age categories in order to provide athletes with an opportunity to participate and compete against others who are relatively close to their age. The intended principle is to establish a fair playing field and to increase sport participation which is associated with the promotion of physical, psychological and social well-being among middle and older aged adults [9]. However, in recent years, evidence has emerged suggesting there may be problems with the current age categorization practices, indicating that a constituent year effect may be influencing the participation of masters athletes in adult athletics and swimming [10,11].

The constituent year effect, previously termed relative age effect in masters sport [9], pertains to the participation- and performance–related advantage of being relatively younger within a standard age category bracket [9,10]. This phenomenon was first reported in the masters sporting contexts of swimming and athletics in a retrospective study conducted by Medic and colleagues in 2007 [11]. Typical masters-level sports widely utilize competitive age categories which span five years, and thus researchers have utilized them to identify relatively younger and relatively older individuals within distinct five-year brackets. An analysis of participation entries at the United States (US) Masters track-and-field (between 1996 and 2005) and swimming championships (between 1998 and 2005) found that the likelihood of participating in a US National masters championship was lowest when athletes were in the fourth and fifth year of an age category, and highest when they were in the first and second year [9]. In a broader review of relative age effects in sport [10], it was recommended that effects related such relative age disparities in Masters sport be referred to as the constituent year effect, to differentiate it from relative age processes among youth. 

In 2009, research on the constituent year effect in Masters sport was extended to investigate whether similar results would manifest in sports other than athletics and swimming, and to athletes from countries outside of the US. Masters weightlifting and rowing were chosen because competitions in these sports are arranged by age and weight (rather than age only as in Masters track and field and swimming), thus offering an opportunity to examine whether weight categories buffer the potential advantages afforded to relatively younger Masters athletes in any five-year age category. The results of one study [12] indicated that an effect existed at the international level in masters swimming and track and field, but not in weightlifting and rowing, suggesting that the effect could be contingent on the sport in which an adult athlete competes. Specifically it was found that participation in a world-level swimming and track and field competition was significantly higher for individuals participating in their first and second year, and lower in the fourth and fifth year of an age category [12]. Even though there were decreasing tendencies over constituent years, lack of significant effect in Masters weightlifting and rowing was likely because these sports are categorized by both age and weight class and/or due to the lesser popularity (i.e., lower sample size) and cultural importance of these sports. Another study [9] determined the impact of gender, age, and type of sport on the constituent year effect in masters sport. It was found that a participation-related constituent year effect existed in both male and female masters athletes, however, it was stronger for males. Furthermore, participation-related constituent year effects existed during the fourth decade of life and onwards, but not for the third decade of life.

In 2011, a retrospective longitudinal study design examined the participation rates of masters swimmers whose participation was followed from 2003 to 2009 at the US Masters Short Course National Championships as a function of each individual’s constituent year within any five-year age category [13]. Results showed the odds of participating in the championships were enhanced two-fold during the first constituent year of any five-year age category compared to the odds of an athlete participating during the fifth constituent year.

As previous studies relating to constituent year effect in masters athletes have been conducted with a primarily North American demographic, the aim of this study was to explore whether a similar effect exists within the context of Australian masters track and field athletes. It is surprising that Australian data has yet to be examined, especially considering the popularity of adult sport in Australia, the broader dialogue of lifelong sport and sport lifestyles in Australian culture compared to other Westernized nations, and the emergence of adult sport participation in physical activity and health policy discourse in the country [14]. We calculated the participation rate of all competitors and differentiated between males and females and across decades of life in order to determine if the participation-related constituent year effect among masters athletes is influenced by gender and age, respectively. Based on previous studies [9,11,12,13], we hypothesized that constituent year effects will exist in Australian masters athletes, that it will be stronger in men than women, and that it will become progressively stronger across decades of life.

## 2. Methods

### 2.1. Data collection 

Archived data for the results of the National track and field championships between the years 2000 and 2014 were acquired from the Australian Masters Athletics official Internet website [15]. The information gathered from these proceedings were the first and last name of the participants, gender and each athlete’s age at the time of competition (*n* = 6492 entries). Data from the 2006 track and field championships were disregarded, as the competitors’ individual ages were not listed in the proceedings for this year. The age of entrants ranged from 30 years to 101 years.

### 2.2. Procedures

Individual ages for each competitive entry were scored as frequencies in one of five different categories ranging over five constituent years labelled from ‘Year 1’ to ‘Year 5’ (e.g. 30–34, 35–39, 40–44, 45–49, etc.). For example, individuals falling into the ‘Year 1’ category were in the first year of any five-year age category (30, 35, 40, 45, etc.). The same process was followed for ‘Year 2’ (31, 36, 41, 46, etc.), ‘Year 3’ (32, 37, 42, 47, etc.), ‘Year 4’ (33, 38, 43, 48, etc.), and ‘Year 5’ (34, 39, 44, 49, etc.). In order to examine whether a participation-related constituent year effect is influenced by age, participants were divided into age groups based on decades of life (third decade, fourth decade, etc.) as suggested by previous research [9,16]. Considering the small sample size of masters athletes who were in their 90s and 100s when they participated, these individuals were included in the “eighth + Decade” age group.

### 2.3. Data Analysis

Basic descriptive statistics were used to calculate frequency counts and percentages. Differences between expected and observed counts for each of the constituent years within the five-year-age categories were examined using Chi-square goodness-of-fit tests. Finally, in cases where *p* < 0.05, standardised effect sizes were calculated to evaluate the magnitude of a participation-related constituent year effect for each constituent year within the five-year age category. According to the guidelines [17] and in line with previous studies [9], the effect size was calculated as the ratio between the observed and the expected frequency and the standard deviation.

## 3. Results

Table 1 presents frequency counts distributions for participation entries in each constituent year of the collapsed five–year age category distribution for an overall sample, as well as across gender and decade of life separately. The results for a total sample of Australian masters track and field athletes indicated the existence of a participation-related constituent year effect (*χ^2^_4_* = 55.31, *p* < 0.0001). Specifically, it was found that significantly more masters athletes competed in the Australian masters track and field championships if they were in the first or second year of an age category and significantly fewer in the fourth and fifth year of an age category. The effect sizes were largest during the first and fifth year of an age category.

**Figure 1:**
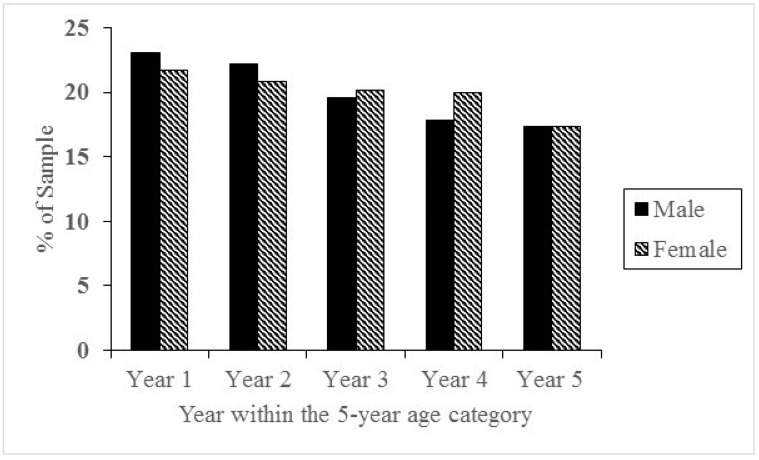
Percentages of masters athletes who competed at Australian Masters Athletics competitions across gender.

The results for gender indicated that a participation-related constituent year effect existed among both male (*χ^2^_4_* = 61.60, *p* < 0.0001) and female (*χ^2^_4_* = 12.67, *p* < 0.001) masters athletes. Results also suggest that significantly more male masters athletes competed in the Australian masters track and field championships in the first two years of an age category and significantly fewer in the last two years. For females, participation was lowest when they were in their last year of an age category. As seen from Figure 1, the participation-related constituent year effect seemed to be stronger for males than females, with more females participating in the third and fourth years of an age category compared to men. For males, the effect sizes were largest during the first and last year of an age category, whereas for females the effect size was largest during last year of an age category.

**Figure 2:**
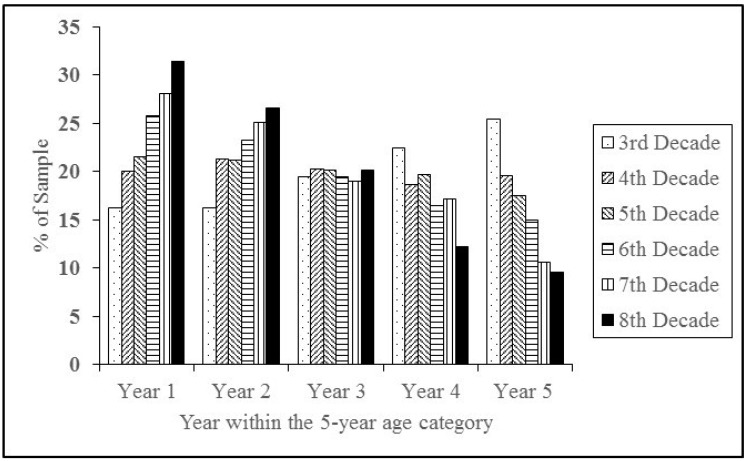
Percentages of masters athletes who competed at Australian Masters Athletics competitions as a function of constituent year across age.

The results across age (Figure 2) indicated that a participation-related constituent year effect in Australian masters athletics does not exist during the fourth (*χ^2^_4_* = 2.91, *p =* 0.573) and fifth (*χ^2^_4_* = 8.86, *p* = 0.065) decade of life, however it was found that it does exist during the third decade of life (*χ^2^_4_* = 24.26, *p* < 0.0001), as well as during sixth decade (*χ^2^_4_* = 24.26, *p* < 0.0001), seventh decade (*χ^2^_4_* = 73.28, *p* < 0.0001), and eighth + decade of life (*χ^2^_4_* = 32.16, *p* < 0.0001). Specifically, an inverse participation-related constituent year effect exists in the third decade such that the likelihood of participating in the first and second year of an age category (i.e., 35 or 36 years) was lower, whereas the likelihood of participating during the fifth year of an age category (i.e., 39 years) was higher. Also, as individuals progress into the sixth decade of life onwards, the likelihood of participating in masters athletics becomes significantly higher if one is in the early part (i.e., first or second year) of an age category and lower if one is in the later part (i.e., fourth or fifth year) of an age category.

## 4. Discussion

In the present study, we sought to replicate and extend previous findings on the constituent year effect in masters sport by utilizing data on masters athletes from Australia. Thus, this study was designed to intentionally fill a void in the existing literature by analysing whether or not a constituent year effect exists within the context of Australian masters track and field. Consistent with previous findings [9,11,12,13], the results indicated that a participation-related constituent year effect is evident overall; specifically that relatively younger masters athletes (i.e., athletes in their first or second year of an age category) participated significantly more often than athletes in their fourth or fifth year of an age category. For the total sample, the effect sizes were largest during the first and the fifth constituent year suggesting that masters athletes were most likely to attend completions when they were relatively youngest and avoid attending when they were relatively oldest.

The participation-related constituent year effect was also observed in both males and females, but seemed to be stronger in males. For males, the effect sizes were largest during first and fifth year of an age category, whereas for females the effect size was largest during the fifth year of an age category suggesting that participation at Australian National athletics competitions was lowest during when athletes were in their latest stage of an age category regardless of gender. Consistent with previous studies with masters track and field athletes [9] which have found that the participation-related constituent year effect is stronger in males than females and those in youth setting on relative age effect [18], this could potentially be due to a lower sporting population size of female athletes. With less female competitors [19], the competitive ‘pool’ would be smaller and less competitive [4,8,20,21] than men’s, making it more likely for a female athlete to win an award compared to their male counterparts. It has also been hypothesized that male athletes may be more likely to be concerned with winning than their female counterparts, deriving more meaning from the outcome of the competition and that commitment to sport may vary across gender [9,21,22,23]. This would result in a potentially more ego-oriented, less self-determined motivational profile, leading to a potential decrease in participation in competitions where the possibility of performing well was diminished.

In terms of age as a moderating factor, participation-related constituent year effect was evident in the third decade of life, whereas in previous studies no effect has been observed until at least the fourth decade [9]. However, not only did the constituent year effect exist during the third decade of life in this Australian sample, it was found to be reversed, meaning that there were significantly more masters participants competing in the fifth year of age brackets as opposed to the first and 2nd, with the effect size strongest during the fifth year. This may suggest that during the third decade of life being relatively older in comparison to peers may be advantageous. An alternative possibility is that these constituent year trends are an indicator of the ages when adults begin to return to competition at National Masters championships after having taken time off, following dis-engagement from youth or young adult participation (e.g., in one’s early 20s). Previous studies have shown that a participation-related constituent year effect occurs in masters sports from the fourth decade of life and onwards and becomes more pronounced with age [9]. The results of this study suggested that participation-related constituent year effect was not significant during the fourth and fifth decades, appearing again in the sixth, seventh and eighth + decades and becoming more pronounced with age. The statistical analyses showed that in decades six through eight and above there was greater participation in the first two years of a competitive age category and less participation in the fourth and fifth years of an age category. The effect sizes were largest during first and fifth year of an age category implying that Australian masters athletes who are in their 60s and beyond are most likely to attend National athletics competitions when they are relatively youngest and to avoid participating in these competitions when they are relatively oldest within their age groups. These findings are consistent with the those from the previous literature [9] with the exception of the fourth and fifth decades, where a constituent year effect has previously been observed within North American masters swimmers and track and field athletes [9,11,13]. One of the explanations of why participation related constituent year effect gets stronger with age could be explained by physiological factors [24] associated with inevitable declines in athletic performance with age. For example, previous studies suggest that masters athletes in the younger age-groups perform better than the athletes in the older age-groups [4] and that the age decline in running velocity did not occur until the late 50s age group [19]. Also, studies have demonstrated that masters athletes improved their athletic performance significantly and progressively over the years such that the magnitude of improvements was greater in older age groups [25]. In addition, these finding may be related to individual’s perceptions of physical disadvantage of being relatively older, which can also be associated with age-related frailty and mortality. In other words, a difference in performance over a five year period is exemplified during 80s in comparison to 40s (e.g., if one compares a world record in any track and field event, the difference between 40 and 44 years old will be smaller than that of an 80 and 84 years old). Therefore, a relatively older masters athlete who is in his/her 80s may be more likely to have a greater sense of a disadvantage (and thus be less likely to attend competitions) in comparison to a relatively older masters athletes who is in his/her 40s. Another possibility is that older masters athletes (those aged in their 60s and beyond) may be more extrinsically motivated than those in their 30s and 40s. It would also be valuable for future studies to examine the motivations for participation, and to investigate whether a perceived advantage occurs in masters athletes and if it varies across decades of life. Further research might endeavour to look at potential reasons as to why the effect occurs, and why it is more exaggerated in the later decades. Furthermore, there is the possibility that athletes’ decisions to competitively participate as a function of constituent year may interact with event and sport type, which needs to be addressed in future research. For example, within athletics, it would be interesting to observe whether constituent year effects manifest for jumps, running, and technical events equally. It is possible that the competitive pool talent varies for each event type, which may have a bearing on the constituent year effect.

One of the limitations of this study was the cross-sectional nature of the data. Longitudinal studies [13] would better examine the dynamics of within-individual decisions to participate in relation to the constituent years of any five-year age category. Also, in addition to focusing solely on participation rates of Australian masters athletes, future studies could also examine variations in athlete’s performance (rather than solely participation rates) across the constituent years of a five-year age category [26]. Furthermore, given the well documented variations in physical demands [19,20], the age of peak performance [7,8], and competition density [4] within various track and field disciplines (e.g., jumps vs. throws vs. running vs. pentathlon), future studies should examine participation- and performance-related constituent year effect across disciplines of track and field.

These findings are important as they provide further evidence of the existence of the participation-related constituent year effect in more diverse contexts (i.e., Australia) within the masters sport of athletics. In addition, the evidence from this study suggests that the current five-year age categories may not provide an equal competitive opportunity especially for relatively older athletes as for those who are relatively younger, and this is especially the case for males and athletes who are in their later decades of life. It would be valuable that future research focuses on examining other nations (i.e., Europe, Asia, etc.), as well as considering potential solutions for the constituent year effect in order to prevent unintended disadvantages and drop-out in masters athletes. Finally, the awareness of the constituent year effect in masters athletes is not well understood, even though preliminary evidence suggests that some masters athletes are aware of it [27]. It is particularly important that constituent year effect is understood by all those involved in masters sports (i.e., athletes, coaches and administrators) as this can likely affect participants’ motivation and the likelihood of them maintaining training and competition routines. In addition, these intermittent patterns of participation in masters athletes, are likely based, in part, on individual’s perceptions of competency and/or expectancies, and not exclusively related to drop-out due to injury or morbidity. If the five-year age brackets pose challenges to competitive and achievement motivation of adult athletes, it is important for sport programmers and coaches to understand this disruption in order to help athletes navigate and persist in the inevitable face of age-related decline [28]. If these constituent year effects disparities in competitive participation, viewed cross-sectionally in the current study from age 60 onwards, serve as a disruption to continued participation, then the phenomenon becomes a challenge to lifelong participation for competitive enthusiasts. The irony that the five-year brackets were created to ensure continued involvement, yet competitively oriented people may steer away from participation late in a five-year age bracket to never return, is a problem that requires further investigation. Should the constituent year effect in masters sport disrupt continued participation among Australian sport enthusiasts, sport organizers may wish to seek better organizational strategies to continue to shepherd participants forward as they age.

## Figures and Tables

**Table 1:** Participation rate distributions among masters athletes across gender and decade of life.

		Year	Observed Frequency	Expected Frequency	*χ^2^*	*p*	ES
Total Sample		1	1466	1298.4	21.63	<0.0001	1.19
		2	1409	1298.4	9.42	<0.01	0.78
		3	1287	1298.4	0.10	N.S.	-
		4	1206	1298.4	6.58	<0.05	0.66
		5	1124	1298.4	23.43	<0.0001	1.24
Gender	Males	1	966	837.6	19.68	<0.0001	1.19
		2	930	837.6	10.19	<0.01	0.86
		3	821	837.6	0.33	N.S.	-
		4	745	837.6	10.24	<0.01	0.86
		5	726	837.6	14.87	<0.001	1.04
	Females	1	500	460.8	3.33	N.S.	-
		2	479	460.8	0.72	N.S.	-
		3	466	460.8	0.06	N.S.	-
		4	461	460.8	0.00	N.S.	-
		5	398	460.8	8.56	<0.01	1.64
Age	Third decade	1	124	151.8	5.09	<0.05	0.92
		2	123	151.8	5.46	<0.05	0.95
		3	148	151.8	0.10	N.S.	-
		4	171	151.8	2.43	N.S.	-
		5	193	151.8	11.18	<0.001	1.36
	Fourth decade	1	307	306.4	0.00	N.S.	-
		2	327	306.4	1.38	N.S.	-
		3	311	306.4	0.07	N.S.	-
		4	286	306.4	1.36	N.S.	-
		5	301	306.4	0.10	N.S.	-
	Fifth decade	1	384	357.8	1.92	N.S.	-
		2	379	357.8	1.26	N.S.	-
		3	360	357.8	0.01	N.S.	-
		4	353	357.8	0.06	N.S.	-
		5	313	357.8	5.61	N.S.	-
	Sixth decade	1	374	289.4	24.73	<0.0001	1.29
		2	335	289.4	7.19	<0.01	0.70
		3	282	289.4	0.19	N.S.	-
		4	239	289.4	8.78	<0.01	0.77
		5	217	289.4	18.11	<0.0001	1.11
	Seventh decade	1	218	155.4	25.22	<0.0001	1.17
		2	195	155.4	10.09	<0.01	0.74
		3	148	155.4	0.35	N.S.	-
		4	134	155.4	2.95	N.S.	-
		5	82	155.4	34.67	<0.0001	1.38
	Eighth + decade	1	59	37.6	12.18	< 0.001	1.23
		2	50	37.6	4.09	< 0.05	0.71
		3	38	37.6	0.00	N.S.	-
		4	23	37.6	5.67	< 0.05	0.84
		5	18	37.6	10.22	< 0.01	1.13

Note. N.S. = non-significant.

## References

[1] Bouchard C., Blair S.N., Haskell W.L. (2012). Physical Activity and Health.

[2] Biddle S.J.H., Fox K.R., Boutcher S.H. (2000). Physical Activity and Psychological Well-Being.

[3] Hirvensalo M., Lintunen T. (2011). Life-course perspective for physical activity and sports participation. Eur. Rev. Aging Phys. Act..

[4] Nikolaidis P.T., Zingg M.A., Knechtle B. (2017). Performance trends in age-group runners from 100 m to marathon—The World Championships from 1975 to 2015. Scand. J. Med. Sci. Sports.

[5] Unterweger C.M., Knechtle B., Nikolaidis P.T., Rosemann T., Rust C.A. (2016). Increased participation and improved performance in age group backstroke master swimmers from 25–29 to 100–104 years at the FINA World Masters Championships from 1986 to 2014. Springerplus.

[6] Young B.W., Medic N., Murphy S. (2012). Expert masters sport performers: Perspectives on age-related processes, skill retention mechanisms, and motives. Oxford Handbook on Sport and Performance Psychology.

[7] Allen S.V., Hopkins W.G. (2015). Age of peak competitive performance of elite athletes: A systematic review. Sports Med..

[8] Elmenshawy A.R., Machin D.R., Tanaka H. (2015). A rise in peak performance age in female athletes. Age.

[9] Medic N., Young B.W., Starkes J.L., Weir P.L., Grove J.R. (2009). Gender, age, and sport differences in relative age effects among US Masters swimming and track and field athletes. J. Sports Sci..

[10] Wattie N., Cobley S., Baker J. (2008). Toward a unified understanding of relative age effects in sport. J. Sports Sci..

[11] Medic N., Starkes J.L., Young B.W. (2007). Examining relative age effects on performance achievement and participation rates in Masters athletes. J. Sports Sci..

[12] Medic N., Starkes J.L., Weir P.L., Young B.W., Grove J.R. (2009). Relative age effect in masters sports: Replication and extension. Res. Q. Exerc. Sport.

[13] Medic N., Young B.W., Medic D. (2011). Participation-related relative age effects in Masters swimming: A 6-year retrospective longitudinal analysis. J. Sports Sci..

[14] Eime R.M., Harvey J.T., Dionigi R.E., Gard M. (2018). Sport participation across the lifespan: Australian trends and policy implications. Sport and Physical Activity across the Lifespan: Critical Perspectives.

[15] Australian Masters Athletics. https://www.australianmastersathletics.org.au.

[16] Roberts B.W., DelVecchio W.F. (2000). The rank-order consistency of personality traits from childhood to old age: A quantitative review of longitudinal studies. Psychol. Bull..

[17] Vincent W.J. (1999). Statistics in Kinesiology.

[18] Vincent J., Glamser F.D. (2006). Gender differences in the relative age effect among US Olympic Development Program youth soccer players. J. Sports Sci..

[19] Hunter S.K., Stevens A.A. (2013). Sex differences in marathon running with advanced age: Physiology or participation?. Med. Sci. Sports Exerc..

[20] Kundert A.M.L., Di Gangi S., Nikolaidis P.T., Knechtle B. (2018). Jumping and throwing performance in the World Masters’ Athletic Championships 1975–2016. Res Sports Med..

[21] Cheuvront S.N., Carter R., Deruisseau K.C., Moffatt R.J. (2005). Running performance differences between men and women: An update. Sports Med..

[22] Deaner R.O. (2013). Distance running as an ideal domain for showing a sex difference in competitiveness. Arch. Sex. Behav..

[23] Wigglesworth J.C., Young B.W., Medic N., Grove J.R. (2012). Examining gender differences in the determinants of Masters Swimmers’ sport commitment. Int. J. Sport Exerc. Psychol..

[24] Tanaka H., Seals D.R. (2008). Endurance exercise performance in Masters athletes: Age-associated changes and underlying physiological mechanisms. J. Physiol..

[25] Akkari A., Machin D., Tanaka H. (2015). Greater progression of athletic performance in older Masters athletes. Age Ageing.

[26] Connick M.J., Beckman E.M., Tweedy S.M. (2015). Relative age affects marathon performance in male and female athletes. J. Sports Sci. Med..

[27] Medic N., Young B.W., Grove J.R. (2013). Perceptions of five-year competitive categories: Model of how relative age influences competitiveness in masters sport. J. Sports Sci. Med..

[28] Young B.W., Callary B., Niedre P.C. (2014). Exploring novel considerations for the coaching of Masters athletes. Int. Sport Coach. J..

